# Differential contribution of between and within‐brain coupling to movement synchronization

**DOI:** 10.1002/hbm.26335

**Published:** 2023-05-17

**Authors:** Inbar Z. Marton‐Alper, Andrey Markus, Michael Nevat, Rotem Bennet, Simone G. Shamay‐Tsoory

**Affiliations:** ^1^ Department of Psychology University of Haifa Haifa Israel; ^2^ The Integrated Brain and Behavior Research Center (IBBRC) University of Haifa Haifa Israel

**Keywords:** hyperscanning, interbrain coupling, movement synchronization

## Abstract

A fundamental characteristic of the human brain that supports behavior is its capacity to create connections between brain regions. A promising approach holds that during social behavior, brain regions not only create connections with other brain regions within a brain, but also coordinate their activity with other brain regions of an interaction partner. Here we ask whether between‐brain and within‐brain coupling contribute differentially to movement synchronization. We focused on coupling between the inferior frontal gyrus (IFG), a brain region associated with the observation‐execution system, and the dorsomedial prefrontal cortex (dmPFC), a region associated with error‐monitoring and prediction. Participants, randomly divided into dyads, were simultaneously scanned with functional near infra‐red spectroscopy (fNIRS) while performing an open‐ended 3D hand movement task consisting of three conditions: back‐to‐back movement, free movement, or intentional synchronization. Results show that behavioral synchrony was higher in the intentional synchrony compared with the back‐to‐back and free movement conditions. Between‐brain coupling in the IFG and dmPFC was evident in the free movement and intentional synchrony conditions but not in the back‐to‐back condition. Importantly, between‐brain coupling was found to positively predict intentional synchrony, while within‐brain coupling was found to predict synchronization during free movement. These results indicate that during intentional synchronization, brain organization changes such that between‐brain networks, but not within‐brain connections, contribute to successful communication, pointing to shift from a within‐brain feedback loop to a two‐brains feedback loop.

## Graphical abstract

To explore whether inter‐brain and intra‐brain coupling contribute differentially to movement synchronization, we examined coupling between the inferior frontal gyrus and the dorsomedial prefrontal cortex within and between brains, while participants were performing an open‐ended 3D hand movement task. Inter‐brain coupling in the IFG and dmPFC was evident in the free movement and intentional synchrony conditions but not in the back‐to‐back condition. Importantly, inter‐brain coupling was found to positively predict intentional synchrony, while intra‐brain coupling was found to predict synchronization during free movement.

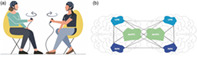

## 1 INTRODUCTION

Extant research proposes that functional brain networks reconfigure in response to changes in one's environment (Fries, 2005). In recent years, the network approach has been expanded to consider brains of interacting individuals as components of a between‐brain network that dynamically changes during social interactions (Dikker et al., 2017; Shamay‐Tsoory, 2021). These functional between‐brain networks emerge from coordinated activity of brain regions of two or more individuals participating in joint action, ranging from face‐to‐face dialogue (Jiang et al., 2012) to interactive song learning (Pan et al., 2018), or cooperation (Cui et al., 2012). While it is becoming well established that between‐brain coupling contributes to social connectedness and is not merely an epiphenomenon of shared sensorimotor context (Gugnowska et al., 2022), the question remains whether between‐ and within‐functional brain networks contribute differently to successful social interactions.

The current study aimed to examine this question by probing between‐brain and within‐brain coupling during a movement synchronization paradigm. Interpersonal synchrony requires coordination of movement over time of two or more interacting individuals (Chartrand & Lakin, 2013; Delaherche et al., 2012). Recent theories of social alignment view movement synchronization as a behavior linked to “higher” levels of alignment including emotional alignment and cognitive alignment (Rossignac‐Milon & Higgins, 2018; Shamay‐Tsoory et al., 2019). For example, studies show that manipulating facial movement affects emotional contagion (e.g., Hawk et al., 2012). Likewise, movement synchronization during psychotherapy was shown to predict therapeutic relationship (Ramseyer & Tschacher, 2011). These studies indicate that movement synchronization is related to other forms of emotional and cognitive alignment and that this behavior may represent a basic form of interpersonal connectedness.

Synchronization frequently emerges spontaneously, when there is no explicit social goal of alignment, as evident in unintentional synchronization of body gestures and facial expressions (Chartrand et al., 2012; Dimberg et al., 2000) synchronized clapping (Néda et al., 2000) and walking (Zivotofsky & Hausdorff, 2007). Notably, several types of behavioral alignment in humans, such as soldiers marching in step (McNeill, 1995), collective religious rituals (Brown, 1922), dances, or musical performances (Cross, 2009) are intentional, pre‐planned, and may require training, pointing to the potential contribution of collective behavior to human socialization (Wiltermuth & Heath, 2009). While the dynamics of spontaneous synchrony resemble that of intentional synchrony (Richardson et al., 2007), it was suggested that in intentional synchrony levels of synchronization are high as it involves explicit monitoring of one's actions in comparison to the interaction partner's actions (Marton‐Alper et al., 2020).

As attaining synchronization requires making predictions regarding the other's movement as well as continuous observation and updating of predictions (Wolpert et al., 2003), it necessitates brain systems that (1) monitor the gap (error) between oneself and the interaction partner, (2) observe the partner's action, and automatically activate one's own representations of this behavior.

Error monitoring has been linked to brain activity of the anterior cingulate cortex (ACC), dorsal medial prefrontal cortex (dmPFC), and insula (Carter et al., 1999; Menon et al., 2001). Interpersonal synchrony was also found to involve temporal coordination that includes temporal prediction and error monitoring (Cacioppo et al., 2014). Notably, single‐brain fMRI study examining the neural substrates underlying dynamic joint action found that in addition to the observation–execution system, brain areas associated with error‐detection and correction such as the dmPFC and the anterior insula were positively correlated with increased perceived synchronization difficulty (Fairhurst et al., 2013).

In addition to the error monitoring system, previous neuroimaging studies on synchronization focused on the inferior frontal gyrus (IFG), a core region of observation–executions system, as it is commonly active both when an action is performed and when it is passively observed (Rizzolatti & Craighero, 2004). The IFG was found to play a role in the dynamic coupling of action observation to action execution (Newman‐Norlund et al., 2007) and to be involved in understanding interpersonal action congruency (Shibata et al., 2011). Studies using the hyperscanning approach have found that the IFG is commonly coupled in its activity between interacting partners during social interactions that require coordination (e.g., Funane et al., 2011). Likewise, between‐brain coupling in the IFG was reported in a study on speaker–listener coupling during natural verbal communication (Stephens et al., 2010). Research shows increased between‐brain coupling in the IFG during mutual adaptation (Dai et al., 2018) and an increase in both interpersonal fingertip movement and interbrain neural synchronization of the IFG and ACC was found after cooperative interaction (Yun et al., 2012).

While during intentional synchrony cognitive efforts are primarily invested in attempting to become attuned to others, to minimize errors, during free movement efforts are focused on self‐movement and less on the other's movement (Marton‐Alper et al., 2020). In line with this, a previous study reported diminished neural activity of self‐produced speech during synchronized live speech with others (Jasmin et al., 2016), indicating decrease in self‐referred brain activity during mutual alignment. Thus, it could be the case that between‐brain connectivity in regions associated with alignment (IFG) and gap detection (dmPFC) would be highest when behavior is highly synchronous, because in this case both brains can be expected to activate their respective alignment systems concurrently over time. Conversely, increased within‐brain coupling may be expected in interactions which consist of only sporadic gap detection (such as spontaneous synchrony), which would result in few instances of co‐activation between brains. However, when participants do become aware of such gaps, the higher the amount of corrective action applied by the alignment system within each brain, the more behavioral synchrony would be expected.

Some support for the idea of a potential trade‐off between connectivity of within versus between brain networks may be found in studies characterizing the dynamics of topological brain networks of segregation (high connectivity within specialized networks) and integration (connections between specialized networks; Deco et al., 2015). As evidence points to an inherent trade‐off between functional segregation within networks and integration between brain networks (Deco et al., 2015; Park & Friston, 2013; Tononi et al., 1994), it is possible that there is also a balance in the dynamics of within and between brain networks such that as interaction partners become highly coupled, networks topology changes towards between‐, rather than within‐brain connectivity. It may be thus argued that when there is a gap between the participants, within‐brain coupling between the error and observation–execution network may support the emergence of synchronization. However, when synchronization is high during intentional synchrony, mutual predictions, and increased reliance of the other's action representations is critical (Shamay‐Tsoory, 2021). Therefore, it may be hypothesized that high levels of synchronization should be supported by increased between‐brain coupling rather than within‐brain coupling.

**FIGURE 1:**
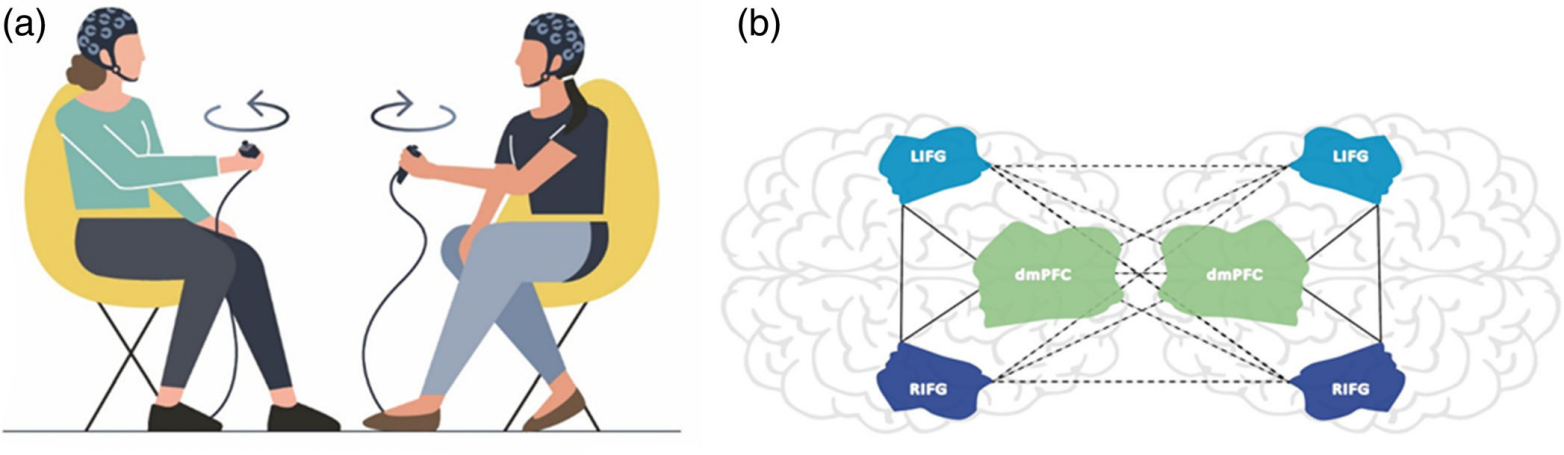
(a) *Task setting*. Participants are seated facing each other. Each participant is holding a Razer Hydra game controller in his/her hand. The device tracks motion and orientation of hands. It is held by the participant's hand and allows any type of movement including circular movements, movement away and towards the body as well as movements to the right and left sides of the body. (b) Measures of between‐brain and intra‐brain coupling. Solid lines represent intra‐brain network and dashed lines represent between‐brain networks. Our model holds that intentional synchronization relies on between‐brain networks rather than within‐brain networks.

Here, our goal was to determine whether interpreting network organization in terms of between‐ and within‐brain networks could explain successful movement synchronization. We scanned dyads with fNIRS while they performed an open‐ended movement task (Reiss et al., 2019; Figure 1a) consisting of a back‐to‐back condition, a free movement condition (low synchrony) and intentional synchrony (high synchrony) conditions. As simple movements, such as button pressing and finger tapping, are mainly characterized by having a distinguishable tempo but do not involve complex motor form or plan, and thus cannot provide a full representation of interpersonal motor synchrony in real‐life, we focused on complex motion in three dimensions (3D) which allows for flexible and diverse movements that require improvisation of dynamic motor planning. 3D motion involves higher activity in the prefrontal structures compared with 2D movement (Milla et al., 2019), demanding a greater cognitive effort. Previous studies have rarely examined brain coupling of interacting participants during interpersonal synchrony within a complex 3D motion environment, even though everyday interpersonal synchrony in tasks such as dancing or playing sports or playing an instrument as a part of ensemble involves varied and complex motor gestures that require motor planning and improvisation. Investigation of 3D motion allows examination of both the form and the speed of elaborate motor actions, thus providing an ecological measurement for examining motor synchrony during interpersonal interactions. To that end, we used a time‐varying measure of dyadic synchrony to examine behavioral synchrony between dyads holding a game controller and instructed to move their hands in 3D space (Reiss et al., 2019), while measuring the within‐ and between‐brain coherence of dyad members using a dual fNIRS system (see link to video of the task^1^). Coupling between two IFGs of interaction partners may support coordination in action representations while coupling between dmPFCs supports mutual predictions. In the case of coupling between two hubs (observation–execution and error detection) between‐brain coupling may support coordination of one's action representation and the other's error detection (Figure 1b). We predicted that overall face‐to‐face interaction, as opposed to back‐to‐back movement, will involve increased within‐ and between‐brain coupling in the error‐monitoring system (dmPFC) and the observation‐execution system (IFG; see Figure 1b). Critically, we predicted that as intentional synchronization requires greater reliance on the other's representation, between‐brain coupling but not intra‐brain coupling would positively predict intentional synchronization.

## 2 METHODS

### 2.1 Participants

The sample consisted of 80 healthy undergraduates from the University of Haifa. A total of 64 women and 16 men (mean age 23.74 ± 4.24) participated in this study for class credit or payment. All participants had normal or corrected‐to‐normal vision. Exclusion criteria included any history of a neurological or psychiatric condition, or chronic illness. All participants were randomly assigned into dyads of the same gender to avoid intergender effects, using an online random number generator.^2^ No gender differences in task performance (measured by CVV, see Section 2.3 for further detail) were observed between the women (*M* = 0.423, SD = 0.021) and the men (*M* = 0.465, SD = 0.044; *F* = 0.735, *p* = .4). Six dyads were excluded either for not completing the required task or due to data acquisition difficulties, leaving data from 34 dyads for analysis. Participants were unaware of the experimental hypothesis prior to participation. All participants provided their written informed consent to participate in the study. The Institutional Review Board (IRB) at the University of Haifa approved the experiment (approval number 032/18), including the written consent procedure.

### 2.2 The Razer 3D synchrony task

The Razer task allowed the assessment of time‐varying interpersonal synchrony between the 3D movements of two individuals using hand‐held Razer Hydra game controllers (Reiss et al., 2019). The Razer hydra device is a game controller that tracks motion and orientation of hands. It is held by the participants and allows free movements in space. Participants were instructed to move their hands and produce any type of movement they want, including circular movements, movement away and towards the body as well as movements to the right and left sides of the body. As seen is the clip (https://youtu.be/VEPWVY9dzRM), movements created frequently were dance‐like or rhythmic and were repeated at regular intervals, forming a regular pattern of direction.

**FIGURE 2:**
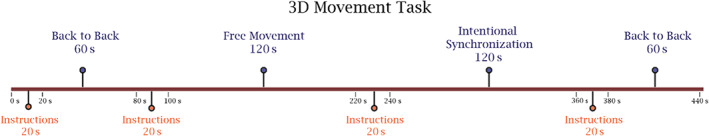
Illustration of the task design.

The task included three conditions (Figure 2): (1) To measure baseline activity when there is no interaction, we included a 2 min of back‐to‐back condition (BB), at the beginning and end of the experiment, whereby participants were seated back‐to‐back and were asked to move their hands freely, as they wished, with no restriction in direction or speed; (2) a free movement condition (FM) in which participants moved their hands freely while facing each other, and (3) an intentional synchrony condition (IS) in which participants were explicitly instructed to move their hands in synchrony. There were no specific instructions other than trying to synchronize their hand movements as much as they can. Each of the task conditions lasted 2 min (120 s) with a 15 s break between conditions. Results of a preliminary pilot study carried out in our lab indicated that intentional synchrony may affect the emergence of spontaneous synchrony during free movement, and therefore all participants in the experiment performed the BB condition first, then the FM condition, followed by the IS condition and then again, the BB condition. Participants were asked to sit still throughout the entire session, avoiding any head or body movement, other than movement of their hands and arms, and to refrain from verbal communication. Participants were posited such that one member of a dyad was facing the other. This meant that motions by one participant's right hand were mirrored by the other's left hand, and vice versa. The use of hand by each given participant was determined randomly.

The controllers recorded the participants' hand position in three spatial axes: *x* represented the position along the line between the participants (forward/backward), *y* represented the perpendicular horizontal axis (right/left), and *z* represented the vertical axis (up/down). All participants were right‐handed. Razer Hydra gaming controllers (Sixense, Los Gatos, CA) were used, which enabled tracking of hand motions at a sampling frequency of 50 Hz.

### 2.3 Task measurements

**FIGURE 3:**
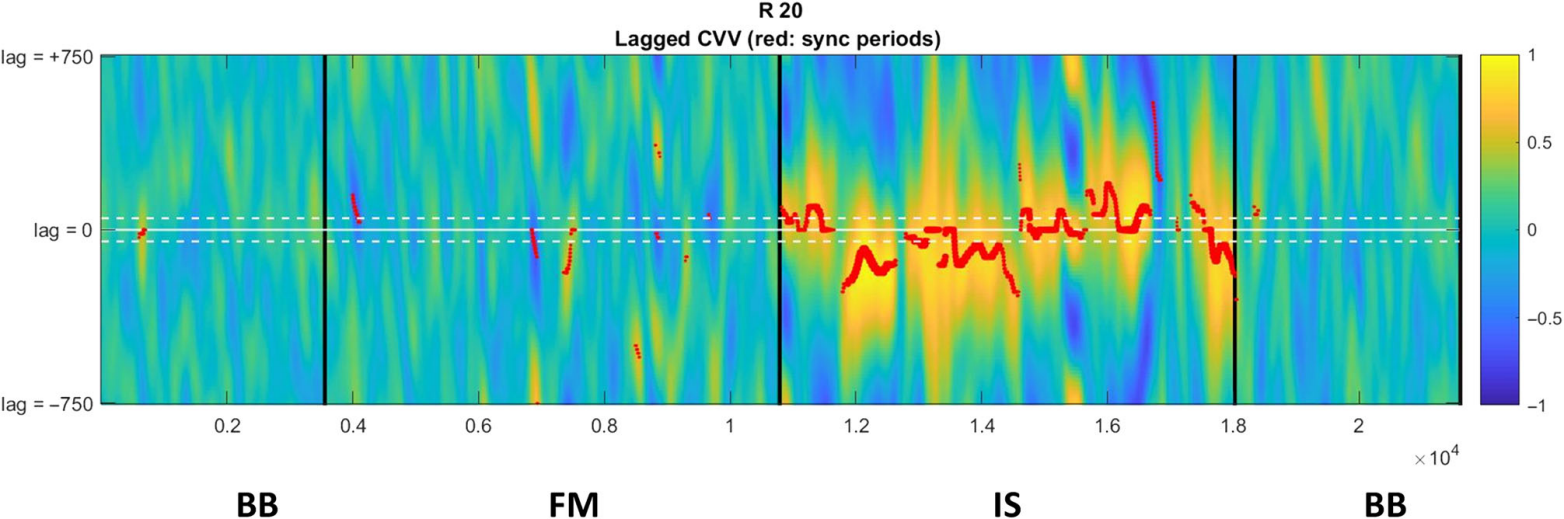
*The level of behavioral synchrony*, encoded by color, of a specific dyad in the different conditions during the Razer task. The *x*‐axis represents time, while the *y*‐axis represents the time lag between participants synchronized motion (i.e., lag 0 indicates no time delay between participants' synchronized movements, lag ± indicates participants performed synchronized motion with time difference). The colors represent the correlation between participants' movements. Colors scaling to deep yellow/blue indicate higher correlation, and thus, greater synchronization between individuals' movements. Red marks indicate sync periods. This graph indicates synchrony is greater during IS condition compared with all other conditions.

Positions of participants' hands in 3D space were recorded for 120 s in each experimental condition, at a sampling rate of 50 Hz. Instantaneous velocities were calculated as the difference between consecutive position measurements for each participant. To eliminate jitter, a 100 ms Gaussian sliding window was applied to the calculated velocities (Figure 3). Synchrony between participants' movements was measured by calculating the cosine of the angle between 3D velocity vectors (CVV) across different time lags (Reiss et al., 2019). This cosine is given by $C_{i,j}\left(t\right)=\frac{<v_{i}\left(t\right),v_{j}\left(t\right)>}{|v_{i}\left(t\right)\|v_{j}\left(t\right)|}$, where t is the time point, <*v*_*i*_ *v*_*j*_> is the inner product of the velocities of the two participants, and |*v*_*i*_| and |*v*_*j*_| are the magnitudes of the velocities of the *i* participant and the *j* participant, respectively. CVV values ranged between 1 and −1, where CVV = 0 suggests there is no alignment in the vector directions, whereas CVV = 1 indicates that the two vectors point in the exact same direction reflecting identical movement, and CVV = −1 indicates that the two vectors point in opposite directions, reflecting mirror‐like movement (e.g., when the participants are either moving toward each other or away from each other). We considered both movement in the same direction and movement in opposite directions to be indicative of synchronization, and therefore used the absolute value |CVV| as our measure of interpersonal synchrony at varying time windows. For each condition (FM, IS) the absolute CVV of each dyad was calculated at each time point (for each time interval of 120 s, a total of 6000 time points at a sampling rate of 50 points per second). Windowed cross‐correlation between the two CVV time‐series was computed for each condition, smoothed over a sliding window size of 100 samples (2 s), and computed for all the lags within a range of ±750 ms (in steps of 10 ms). A threshold of |CVV| > 0.35 was used to identify moments of high motion correlation, that is, highly similar motion, either synchronized or with some lag between the participants. This threshold was used to determine the proportion of time in which behavioral synchrony occurred out of the entire duration of each block in the task, for each dyad separately. The threshold value of |CVV| > 0.35 was based on the results of a pilot study, conducted prior to the current study, with the specific intent to extract the most suitable parameters for CVV computation in the Razer task.

The calculated CVV enabled identifying synchronized motion, calculated with the measure *total sync* which refers to the interpersonal synchrony (CVV) participants displayed when performing synchronized motion both at the exact same time (lag 0) and in a time difference >30 ms. The assessment of synchrony was based on the measure of absolute value of motion velocity correlation (CVV), where higher correlation indicates higher synchrony, as demonstrated in Figure 3. Fisher *z*‐transformation was applied to the absolute value of CVV to normalize their distributions (Chang & Glover, 2010). For analysis purposes we also generated a set of control pairings by a random reshuffling of the participants movement patterns, creating “pseudo dyads.” The pseudo dyads consisted of two participants, each from a different real dyad. We further calculated the behavioral synchrony measure total sync both for the real dyads and for the pseudo dyads. Thus, we generated behavioral synchrony measure that represented actual synchronous events that took place during the task performance (i.e., real dyads performance), as well as a measure representing chance‐level baseline synchrony (i.e., pseudo dyads performance).

We used linear mixed effects (LME) models using the R language, as suggested by Baayen et al. (2008), and the *lme4* package for the R language (Bates et al., 2007), to examine the level of synchrony in the different conditions (BB, FM, and IS). In all instances, synchrony values which exceeded ±2.5 SD from the general mean were excluded (8.46%).

### 2.4 fNIRS data acquisition

**FIGURE 4:**
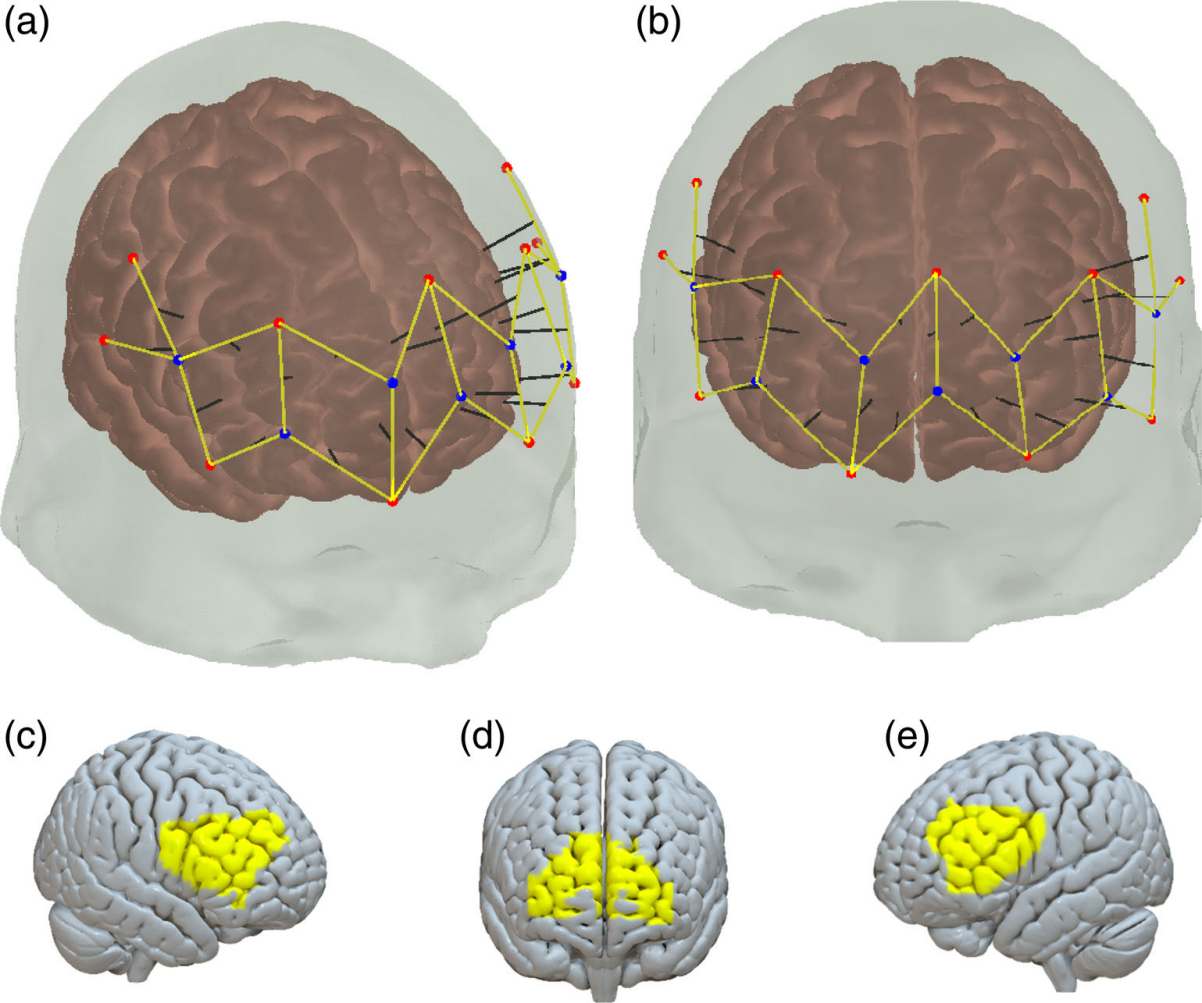
*fNIRS channel placement against anatomical brain areas*: All available channels, right‐side view (a); All available channels, frontal view (b); rIFG coverage (c); dmPFC coverage (d); and lIFG coverage (e). Channels (marked as yellow lines) are formed between Transmitters (red dots), and adjacent receivers (blue dots). The black lines indicate the approximate brain areas corresponding to each channel.

Neural activity from both participants in each dyad was measured simultaneously using two Brite23 functional near infrared spectroscopy (fNIRS) systems (Artinis Medical Systems, Elst, The Netherlands). Each system is comprised of 7 receivers and 11 transmitters resulting in 23 measurement channels per participant, with equal distance of 3.5 cm between optodes (Figure 4). The optodes were positioned on the head according to the international 10–20 system (Okamoto et al., 2004) covering the prefrontal cortices (IFG, dmPFC). Continuous wave fNIRS was used to assess cortical hemodynamic activity using two wavelengths of 760 and 850 nm. Measurements of oxygenated hemoglobin (O^2^Hb) and deoxygenated hemoglobin (HHb) concentrations were obtained at a sampling frequency of 10 Hz using Oxysoft software (v.3.0.52, Artinis Medical Systems, Elst, The Netherlands).

### 2.5 fNIRS data analysis

The MATLAB‐based HOMER2 analysis package (Huppert et al., 2009) was used for preprocessing of the data. A wavelet motion correction procedure was conducted to correct motion artifacts of the optical density data (Molavi & Dumont, 2012). Subsequently, using the modified Beer–Lambert law, optical density data were converted to oxyhemoglobin (O^2^Hb) and deoxyhemoglobin (HbR) concentrations, applying partial volume correction using differential path‐length factor (DPF; Cope et al., 1988). Previous findings show that the direction of changes in O^2^Hb is always an indication for regional cerebral blood flow, whereas the direction of changes in HbR is determined by changes in venous blood oxygenation and volume (Hoshi, 2007). Therefore, we chose to examine O^2^Hb concentration as our measurements for regional cerebral blood flow. Time series of O^2^Hb concentrations in all channels were visually inspected using the HOMER‐graphical interface. Motion‐related artifacts and noisy epochs were excluded from subsequent analyses. Additionally, channels exhibiting excessive noise and channels in which the participants' heartbeat could not be identified were excluded altogether. Following this initial pre‐processing, PCA‐based spatial filtering was applied to the O^2^HB data, as described by Zhang et al. (2016), to correct for scalp blood flow in the fNIRS signals in the absence of short‐separation channels in the Brite23 systems. This method is based on decomposition of the signals; spatial smoothing of the resulting components; subtraction of these smoothed components from the (original) PCA components; and reconstruction of the signals using the differences between the original components and the smoothed components, thereby presumably cleaning global effects. Next, between‐ and within‐brain coupling was estimated by means of a wavelet transform coherence (WTC) function, which enables identification of locally phase‐locked activity, using the wtc‐16 toolbox for Matlab (Grinsted et al., 2004). The average coherence value between 0.15 and 0.015 Hz was calculated. This frequency band allowed us to exclude noise associated with cardiac pulsation (about 1–2 Hz) and respiration (0.2–0.3 Hz). Inter and intra‐brain coupling between each two channels was averaged across each condition (FM, IS) and served as a measure for neural synchrony. Finally, Fisher *z*‐transformation was applied to averaged WTC values to normalize their distributions (Chang & Glover, 2010). In addition, we assessed WTC for unrelated‐paired‐participants (“pseudo dyads”).

Three regions of interest (ROI) were created based on channel localizations and prior knowledge regarding the brain areas associated with social cognition (Shamay‐Tsoory et al., 2019), dividing the channels into prefrontal lateral right and left regions, including two bilateral inferior frontal gyrus (left/right IFG); and the prefrontal medial region including the dorsomedial prefrontal cortex (dmPFC). To examine inter/intra‐brain coherence between participants, we examined WTC values in all pairing of ROIs.

## 3 DATA ANALYSES

We used linear mixed effects (LME) models using the R language, as suggested by Baayen et al., (2008), and the *lme4* package for the R language (Bates et al., 2007), to examine the level of behavioral synchrony in the different conditions (BB, FM, and IS). For the neuroimaging data we also used LME models to examine the level of between and within‐brain coupling in the different conditions (*BB*, *FM*, and *IS*) in pseudo and real groups. To examine whether participants exhibit differences in between‐brain coupling between the various task conditions, we constructed two LME models, which included Condition (BB, FM, and IS), and ROI pairings as fixed factors, dyads' ID numbers as a random factor, and coherence (WTC) values as a dependent measure. To examine brain and behavior relationships we constructed three LME models, each consisting of condition, ROI, and the continuous value of WTC as fixed factors and dyad IDs as a random factor, for prediction of unlagged motion synchrony (“Total Sync”). The specifics of each model are described in the following sections.

## 4 RESULTS

### 4.1 Behavioral synchrony

**FIGURE 5:**
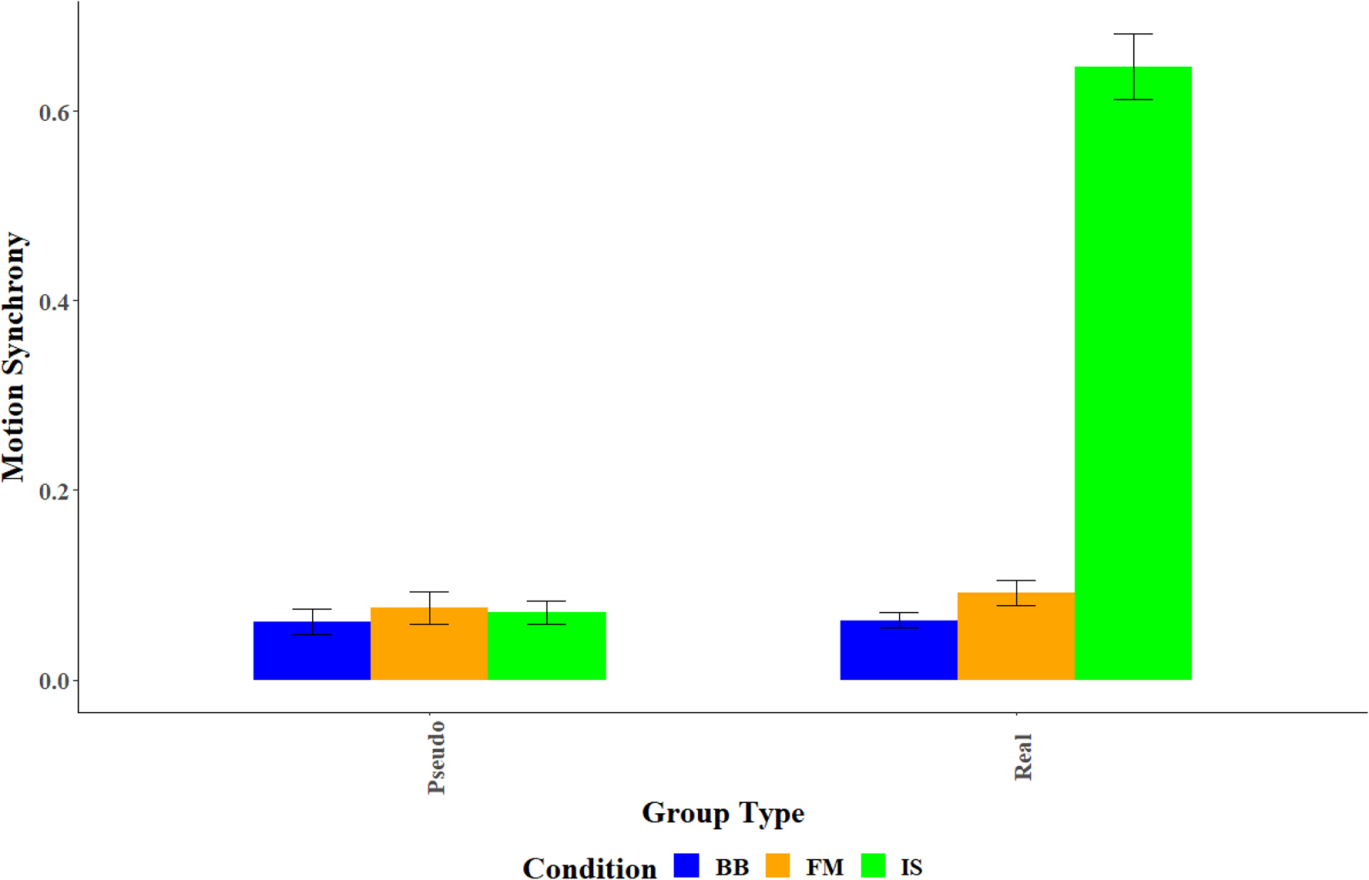
Behavioral synchrony levels between Real and Pseudo group assignment in the BB, IS, and FM conditions, for Total Sync.

To examine the differences in the level of synchrony during real‐time interaction (real groups) versus pseudo dyads, we constructed two LME models. Both included condition (BB, FM, IS) and group assignment (Real, Pseudo) as fixed factors, the dyad's ID number as a random factor, and the total behavioral synchrony as the dependent variable. The two models differed in that the first model took only the main effects of the fixed factors into consideration, whereas the second model also included the interaction between the fixed factors. The increase in the predictive power of the models versus the cost of increasing the complexity of the models due to the interactions between the fixed effects was tested using Type II Wald *χ* ^2^ tests, as per (Baayen et al., 2008). The second model yielded a significantly greater predictive power (*χ* ^2^_(2)_ = 244.51; *p* < .001) and was therefore analyzed further. Analysis of the model revealed a significant interaction between group assignment and condition (*F*_(2,177)_ = 238.26, *p* < .001, *η*_p_^2^ = 0.73). Analyses of this interaction within the model, using Bonferroni correction for multiple comparisons, revealed that in the IS condition, the total behavioral synchrony mean for real groups (*M* = 0.647, SE = 0.163) was significantly greater (*χ* ^2^_(1)_ = 526.41, *p* < .001) than that for the pseudo groups (*M* = 0.07, SD = 0.063). By contrast, no significant differences (*χ* ^2^_(1)_ = 0.47, n.s.) were found in the FM condition between the real (*M* = 0.091, SD = 0.054) and the pseudo (*M* = 0.076, SD = 0.089) dyads. Likewise, no significant differences (*χ* ^2^_(1)_ = 0.01, n.s.) were found in the BB condition between the real (*M* = 0.063, SD = 0.07) and the pseudo (*M* = 0.062, SD = 0.09) dyads—see Figure 5.

### 4.2 Between‐brain coupling

**FIGURE 6:**
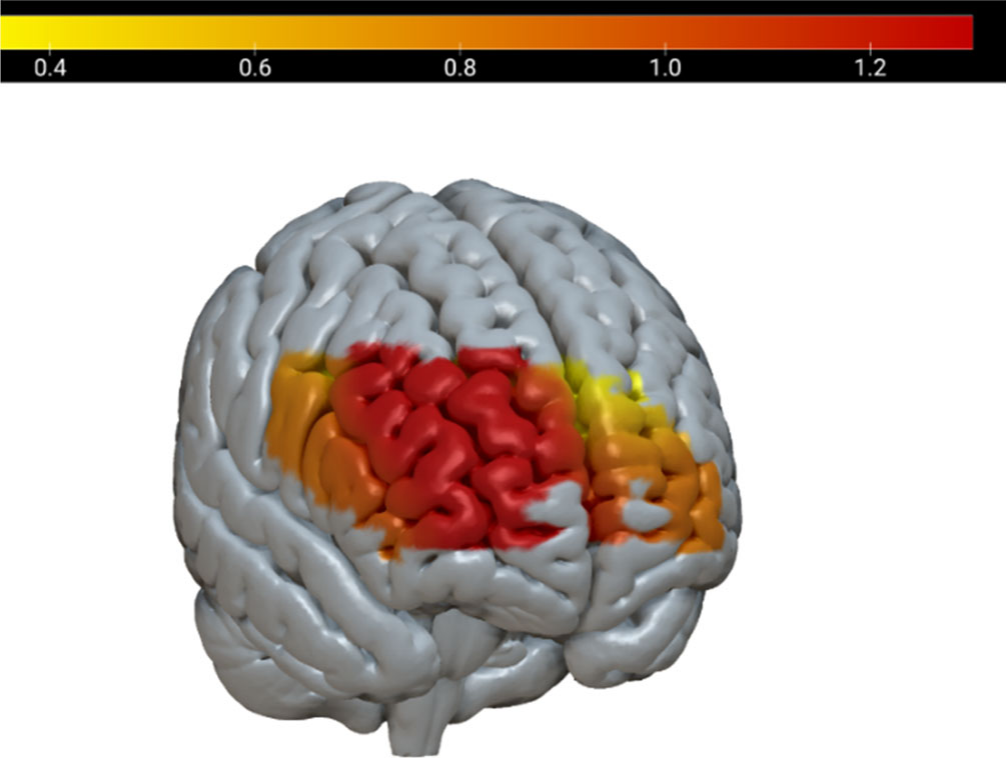
Between‐brain coherence in the R.IFG and dmPFC in the IS condition, showing significant difference from back‐to‐back condition.

**FIGURE 7:**
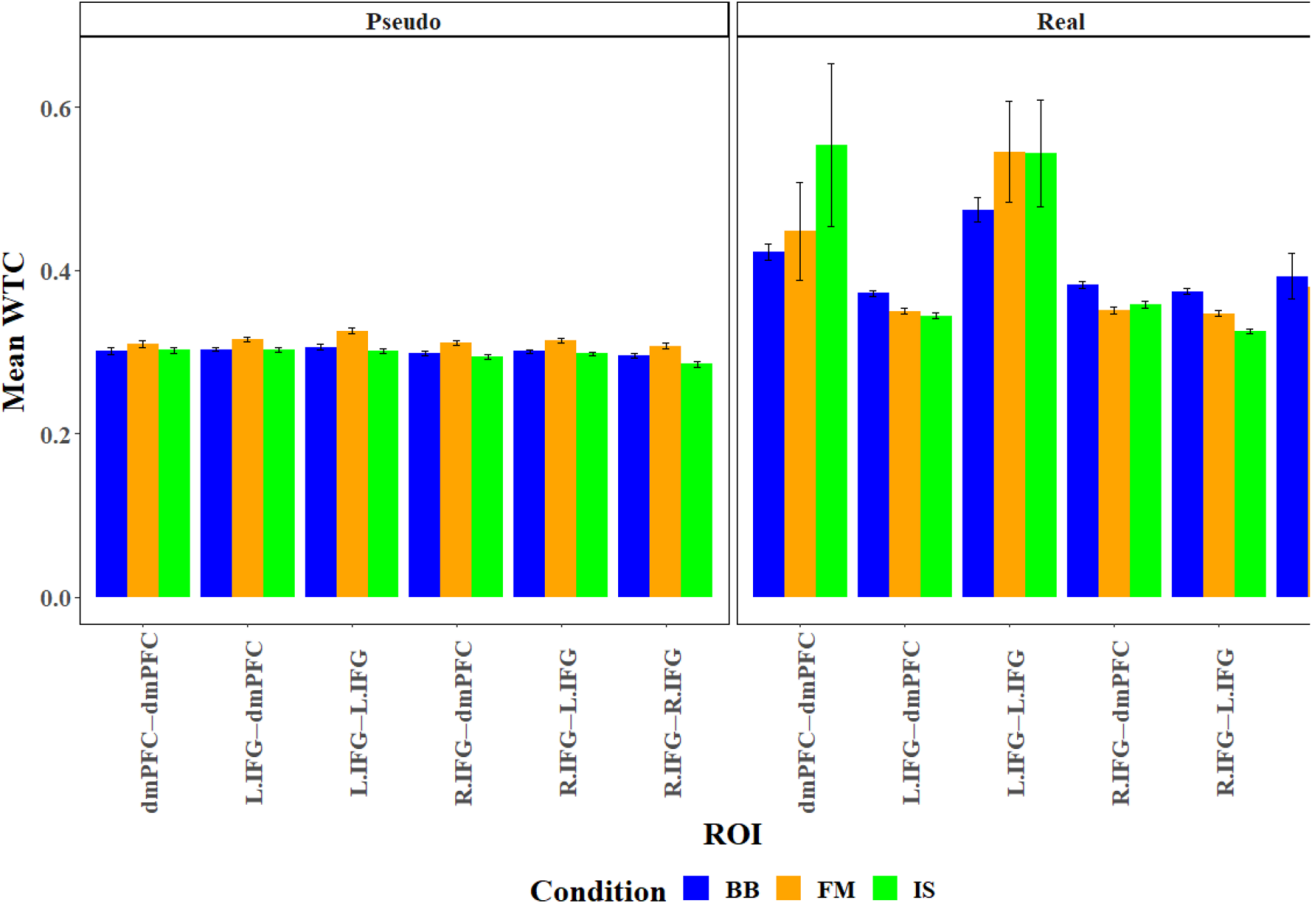
Predicted IBS levels between Real and Pseudo group assignment in the IS and FM conditions.

To examine whether participants exhibit differences in between‐brain coupling when assigned to a real dyad as compared with a pseudo dyad, we constructed three LME models, which included Group Assignment (Pseudo/Real), Condition (BB, FM, and IS), and ROI pairings as fixed factors, dyads' ID numbers as a random factor, and coherence (WTC) values as a dependent measure. The three models differed in their respective levels of interaction between the fixed factors, ranging from main effects only in the first model, through main effects and 2‐way interactions in the second model, to all possible main effects and interactions in the third model. Type II Wald *χ* ^2^ comparison tests showed that the second model yielded a significantly greater predictive power than the first model (*χ* ^2^_(17)_ = 107.67; *p* < .001), and the third model yielded a significantly greater predictive power than the second model (*χ* ^2^_(10)_ = 19.96; *p* < .05). The third model was, therefore, used in further analyses. Analysis of the third model revealed a significant interaction (*F*_(10,26,991)_ = 1.92, *p* < .05, *η*_p_^2^ = 0.001) between Group Assignment, Condition, and ROI, such that in the ROI representing the L.IFG to L.IFG coupling between the participants, coherence values in the FM condition for the Real groups (*M* = 0.546, SD = 1.37) were significantly higher (*χ* ^2^_(1)_ = 16.57; *p* < .001) than those for the Pseudo groups (*M* = 0.326, SD = 0.075); in the IS condition of the Real groups (*M* = 0.544, SD = 1.424) yielded significantly higher coherence values (*χ* ^2^_(1)_ = 21.72; *p* < .001) than those in the Pseudo groups (*M* = 0.302, SD = 0.066); in the BB condition, the Real groups (*M* = 0.474, SD = 0.459) yielded significantly higher coherence values (*χ* ^2^_(1)_ = 16.28; *p* < .001) than those in the Pseudo groups (*M* = 0.306, SD = 0.1). Similarly, in the ROI representing the dmPFC–dmPFC coupling, coherence values in the FM condition for the Real groups (*M* = 0.448, SD = 1) were significantly higher (*χ* ^2^_(1)_ = 11.04; *p* < .05) than those for the Pseudo groups (*M* = 0.31, SD = 0.062); in the IS condition of the Real groups (*M* = 0.553, SD = 1.67) yielded significantly higher coherence values (*χ* ^2^_(1)_ = 15.2; *p* < .005) than those in the Pseudo groups (*M* = 0.303, SD = 0.062); in the BB condition, the Real groups (*M* = 0.423, SD = 0.24) yielded significantly higher coherence values (*χ* ^2^_(1)_ = 10.77; *p* < .05) than those in the Pseudo groups (*M* = 0.302, SD = 0.1). In the ROI representing the R.IFG–R.IFG coupling, coherence values in the IS condition for the Real groups (*M* = 0.494, SD = 1.486) were significantly higher (*χ* ^2^_(1)_ = 10.19; *p* < .05) than those for the Pseudo groups (*M* = 0.285, SD = 0.069; Figure 6). No other significant differences between the Real and Pseudo groups were found, using Bonferroni correction for multiple comparisons. Finally, coherence values in the IS condition, compared with the BB condition was significantly higher for ROIs representing the R.IFG–R.IFG coupling and dmPFC–dmPFC coupling. No other significant differences were found, using Bonferroni correction for multiple comparisons—see Figure 7.

### 4.3 Between‐brain coupling as a behavioral predictor

**FIGURE 8:**
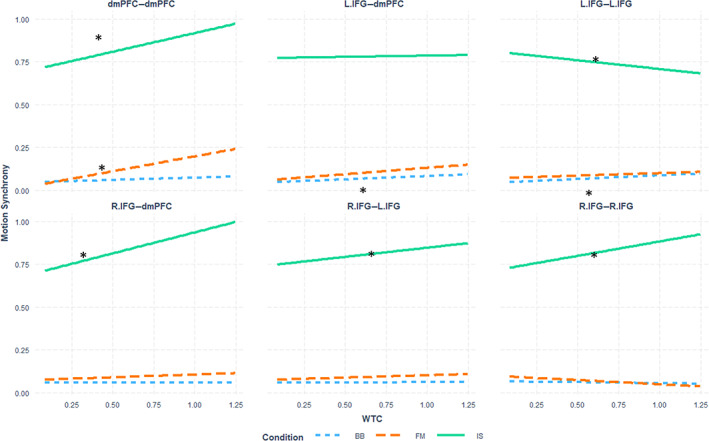
Prediction of Total Sync by WTC in each Condition in each ROI pairing.

To examine the predictive value of between‐brain coupling on behavior, we constructed three LME models, each consisting of Condition, ROI, and the continuous value of WTC as fixed factors and dyad IDs as a random factor, for prediction of unlagged motion synchrony (“Total Sync”). The models differed in the level of interaction between the three fixed effects, such that the first model included only the main effects of the fixed factors, the second model additionally included all 2‐way interactions between the fixed factors, and the third model included main effects and all possible interactions between the fixed factors. A Type II Wald *χ* ^2^ test showed that the second model provided a significantly better prediction compared with the first model (*χ* ^2^_(17)_ = 28.359; *p* < .05), and the third model provided a significantly better prediction relative to the second (*χ* ^2^_(10)_ = 55.01; *p* < .001). Subsequently, the third model, which included all possible interactions between Condition, ROI, and WTC, was used in further analyses in this section (Figure 8). Examination of the LME model showed a significant (*F*_(10,12,873)_ = 5.5, *p* < .001, *η*_p_^2^ = 0.004) interaction between Condition, ROI, and WTC. Since WTC is a continuous predictor, this interaction was examined further using the *interactions* package for the R language. The prediction slopes for Motion Synchrony by WTC within each ROI were examined separately for each Condition (see Figure 8). In the *IS* Condition, WTC was a significant positive predictor of Motion Synchrony values in the dmPFC–dmPFC, L.IFG–L.IFG, R.IFG–dmPFC, R.IFG–L.IFG, and R.IFG–R.IFG ROIs, such that an increase in WTC values predicted an increase in Sync values—see Table 1 for exact slope values. In the *FM* Condition WTC was a significant positive predictor of Sync in dmPFC–dmPFC. In the *BB* Condition WTC was a significant positive predictor of Perfect Sync in L.IFG–dmPFC and L.IFG–L.IFG. Other pairings of ROI by Condition did not yield significant prediction slopes. The values of all the slopes are presented in Table 1.

**TABLE 1:** Numeric values and significance of WTC slopes as predictors of Perfect Sync values in the FM and IS Conditions in each ROI.

	Condition = *IS*	Condition = *FM*	Condition = *BB*
dmPFC–dmPFC	Est. = 0.22 S.E. = 0.07 *t* = 2.93 *p* < .01*	Est. = 0.17 S.E. = 0.06 *t* = 2.77 *p* = .01*	Est. = 0.03 S.E. = 0.03 *t* = 0.97 n.s.
Left IFG–dmPFC	Est. = 0.01 S.E. = 0.04 *t* = 0.34 n.s.	Est. = 0.07 S.E. = 0.04 *t* = 1.73 *p* = .08	Est. = 0.04 S.E. = 0.02 *t* = 2.35 *p* = .02*
Left IFG–Left IFG	Est. = −0.1 S.E. = 0.04 *t* = 2.38 *p* = .02*	Est. = 0.03 S.E. = 0.05 *t* = 0.64 n.s.	Est. = 0.04 S.E. = 0.02 *t* = 2.62 *p* = .02*
Right IFG–dmPFC	Est. = 0.24 S.E. = 0.04 *t* = 5.91 *p* < .01*	Est. = 0.03 S.E. = 0.4 *t* = 0.85 n.s.	Est. = 0 S.E. = 0.02 *t* = 0.06 n.s.
Right IFG–Left IFG	Est. = 0.11 S.E. = 0.03 *t* = 3.24 *p* < .01*	Est. = 0.03 S.E. = 0.03 *t* = 0.82 n.s.	Est. = 0 S.E. = 0.01 *t* = 0.1 n.s.
Right IFG–Right IFG	Est. = 0.17 S.E. = 0.04 *t* = 3.96 *p* < .01*	Est. = −0.05 S.E. = 0.05 *t* = 0.98 n.s.	Est. = −0.01 S.E. = 0.02 *t* = 0.64 n.s.

### 4.4 Within‐brain coupling

**FIGURE 9:**
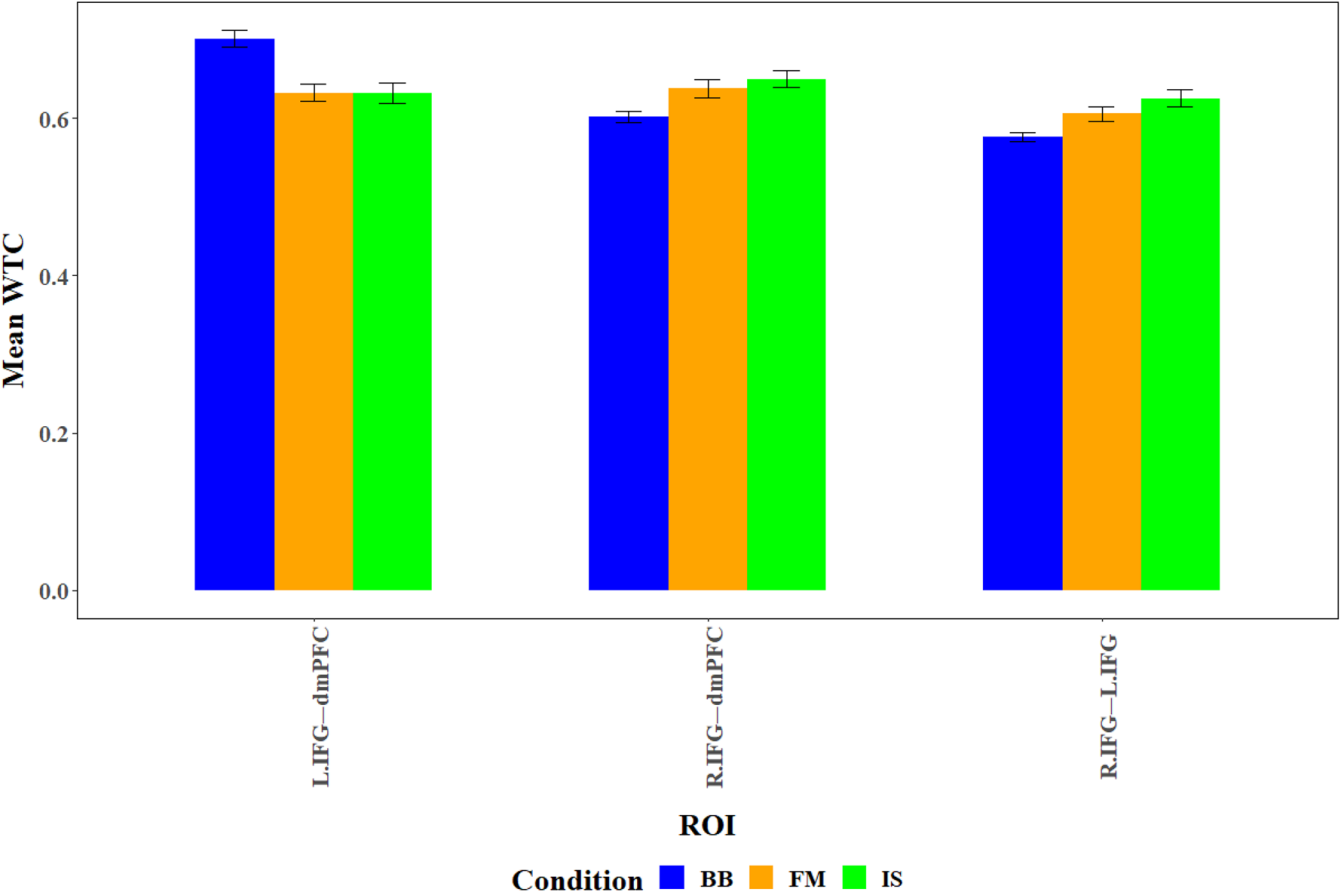
Within‐brain levels between in the BB, IS, and FM conditions.

To examine whether participants exhibit differences in within‐brain coupling between the various task conditions, we constructed two LME models, which included Condition (BB, FM, and IS), and ROI pairings as fixed factors, dyads' ID numbers as a random factor, and coherence (WTC) values as a dependent measure. The first included only the main effects of the fixed factors, while the second model included a possible interaction between them as well. Type II Wald *χ* ^2^ comparison tests showed that the second model yielded a significantly greater predictive power than the first model (*χ* ^2^_(4)_ = 58.61; *p* < .001), and was therefore used in further analyses. Analysis of the second model revealed a significant interaction (*F*_(4,9064)_ = 14.69, *p* < .001, *η*_p_^2^ = 0.006) between Condition and ROI, such that in the R.IFG‐dmPFC ROI coherence in the BB condition (*M* = 0.601, SD = 0.262) was significantly lower than that in the FM (*M* = 0.638, SD = 0.305; *χ* ^2^_(1)_ = 8.34; *p* < .05) and IS (*M* = 0.65, SD = 0.307; *χ* ^2^_(1)_ = 14.79; *p* < .005) conditions, with no significant difference between the FM and IS conditions (*χ* ^2^_(1)_ = 0.69; n.s.). Similarly, in the R.IFG–L.IFG ROI, coherence in the BB condition (*M* = 0.576, SD = 0.252) was marginally lower (*χ* ^2^_(1)_ = 7.49; *p* = .06) than that in the FM condition (*M* = 0.606, SD = 0.287), and significantly lower (*χ* ^2^_(1)_ = 20.5; *p* < .001) than in the IS condition (*M* = 0.625, SD = 0.313). In contrast, in the L.IFG–dmPFC ROI, coherence in the BB condition (*M* = 0.7, SD = 0.383) was significantly higher (*χ* ^2^_(1)_ = 15.33; *p* < .001) than in the FM condition (*M* = 0.632, SD = 0.266), and in the IS condition (*M* = 0.632, SD = 0.3; *χ* ^2^_(1)_ = 20.5; *p* < .001)—see Figure 9.

### 4.5 Within‐brain coupling as a behavioral predictor

**FIGURE 10:**
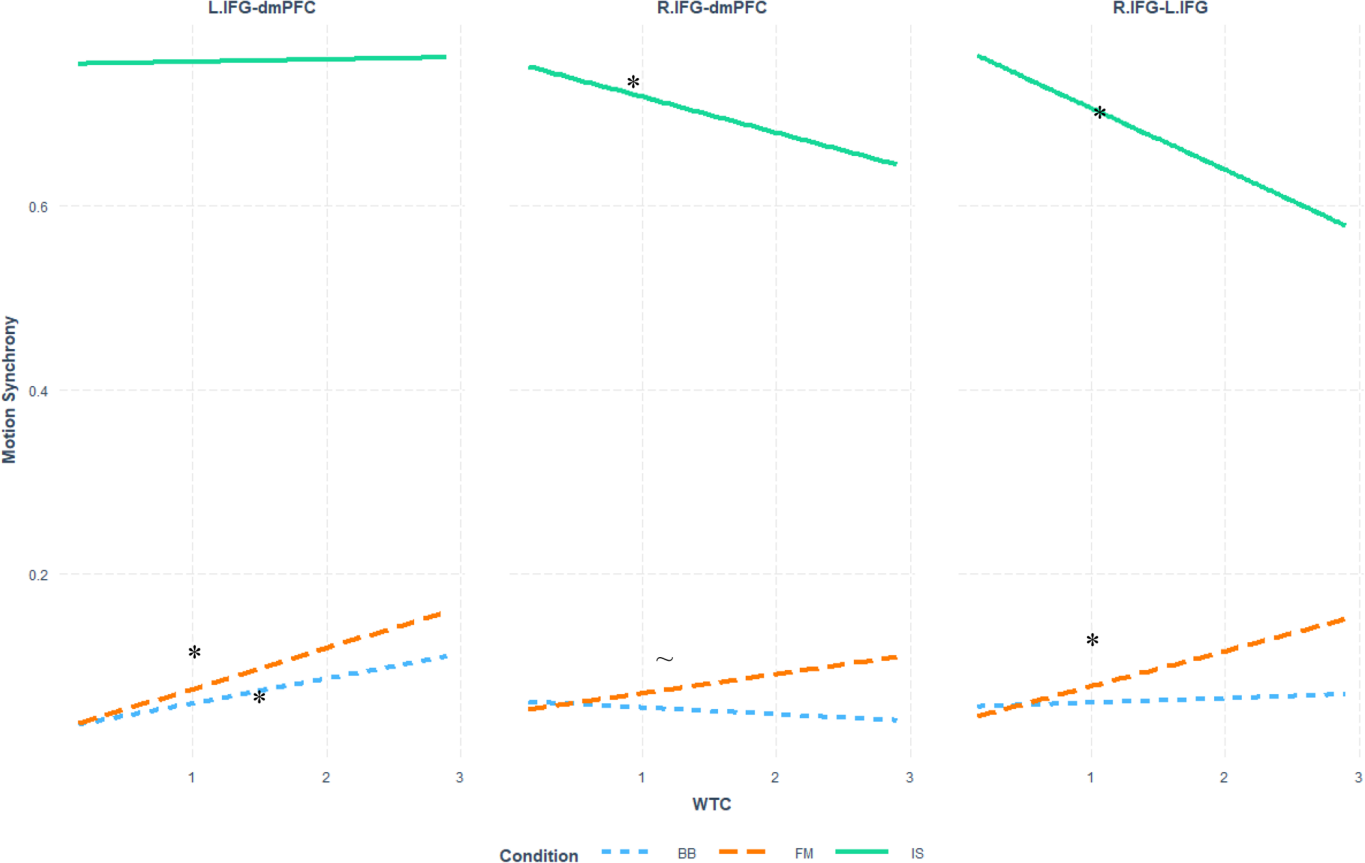
Prediction of Total Sync by within‐brain WTC in each Condition in each ROI pairing.

**FIGURE 11:**
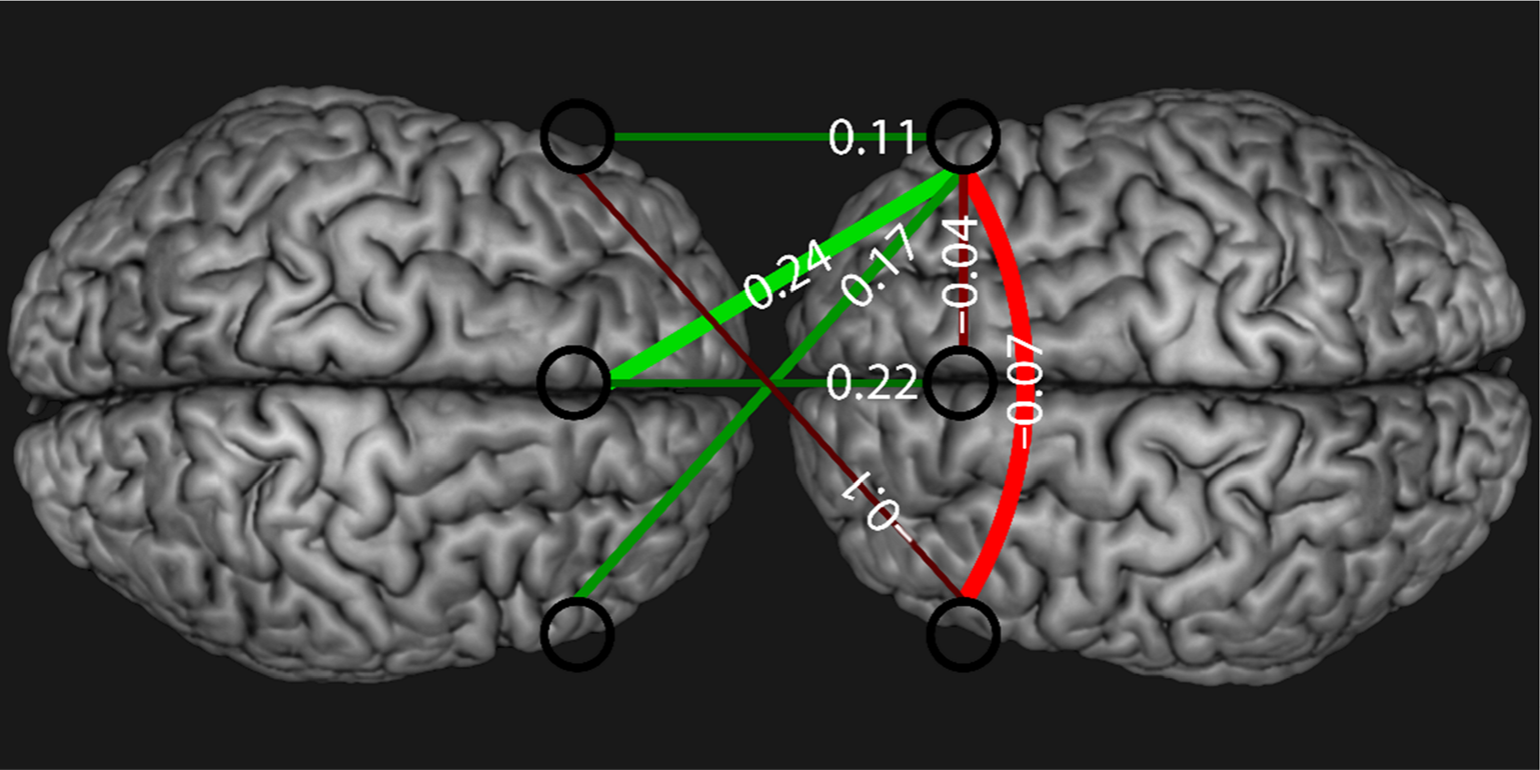
Within‐ and between‐brain coupling strength in the IS condition. Regression *t*‐values are represented by color coding. Positive correlations are in green, negative correlations are in red. The numbers represent regression slope values.

We further examined the predictive value of within‐brain coupling between various brain regions on behavior. In this analysis, we used a subset of three ROIs (L.IFG–dmPFC, R.IFG–dmPFC, and L.IFG–R.IFG), and calculated WTC values within each participant's brain. We constructed three LME models, each consisting of Condition, ROI, and the continuous value of WTC as fixed factors, and subject IDs as a random factor, for prediction of unlagged motion synchrony (“Total Sync”). The models differed in the level of interaction between the three fixed effects, such that the first model included only the main effects of the fixed factors, the second model additionally included all 2‐way interactions between the fixed factors, and the third model included main effects and all possible interactions between the fixed factors. A Type II Wald *χ* ^2^ test showed that the second model provided a significantly better prediction compared with the first model (*χ* ^2^_(8)_ = 119.36; *p* < .001), and that the third model provided a significantly better prediction relative to the second (*χ* ^2^_(4)_ = 10.27; *p* < .05). Subsequently, the third model, which included all interactions between Condition, ROI, and WTC, was used in further analyses in this section. Examination of this model yielded a significant interaction (*F*_(4,9057)_ = 2.5639, *p* < .05, *η*_p_^2^ = 0.001) between Condition, ROI, and WTC coherence. In the L.IFG–dmPFC ROI, WTC coherence was a significant positive predictor of behavioral synchrony in the FM and BB conditions, but not in the IS condition. Notably, in the R.IFG–dmPFC and R.IFG–L.IFG ROIs, WTC coherence was a significant negative predictor of behavioral synchrony only in the IS condition—see Table 2 and Figure 10. A summary of brain and behavior data is presented in Figure 11 which depicts the between and within‐brain predictors of behavioral synchrony in the IS condition.

**TABLE 2:** Numeric values and significance of WTC slopes as predictors of Perfect Sync values in the FM and IS Conditions in each ROI.

	Condition = *IS*	Condition = *FM*	Condition = *BB*
Left IFG–dmPFC	Est. = 0.00 S.E. = 0.01 *t* = 0.19 n.s.	Est. = 0.04 S.E. = 0.02 *t* = 2.92 *p* < .01*	Est. = 0.03 S.E. = 0.01 *t* = 3.72 *p* < .01*
Right IFG–dmPFC	Est. = −0.04 S.E. = 0.01 *t* = 3.40 *p* = .01*	Est. = 0.02 S.E. = 0.01 *t* = 1.81 *p* = .07	Est. = −0.01 S.E. = 0.01 *t* = 0.78 n.s.
Right IFG–Left IFG	Est. = −0.07 S.E. = 0.01 *t* = 6.84 *p* < .01*	Est. = 0.04 S.E. = 0.01 *t* = 3.6 *p* < .01*	Est. = 0.00 S.E. = 0.01 *t* = 0.56 n.s.

## 5 DISCUSSION

With the relatively recent application of brain coherence tools to data collected simultaneously from dyads, there is an emerging interest in understanding how the balance between between‐ and within‐brain networks contribute to effective social interactions. Despite recognition that both within‐ and between brains coupling are critical for social function (Balconi & Fronda, 2021; Santamaria et al., 2020), existing literature relating these two systems across different conditions rarely interprets findings within this between‐ and within‐brain framework. The current study examined how changes between‐ and within‐brain organization relates to interpersonal movement synchronization. The 3D motion paradigm used here allowed us to investigate ecological, complex, and unpredictable movements, which differ from rhythmic and repetitive movements—in particular, from 2D movements restricted to one plane. The paradigm also allowed examining spontaneous synchronization, which emerges at some levels in social interaction involving walking or talking (Zivotofsky & Hausdorff, 2007) as well as intentional synchronization which is required in numerous situations involving dance, choir singing (Müller & Lindenberger, 2011), playing music in ensembles as well as higher intentional forms of alignment, including conformity and cooperation (Levy et al., 1998). The results indicated greater behavioral synchrony during the IS condition compared with the BB and FM conditions, confirming the validity and internal consistency of the conditions. Moreover, when comparing the dyads' performance in the task to “pseudo” interaction, the results show greater interpersonal synchrony between “real dyads” compared with “pseudo dyads” in the IS condition alone, whereas in the FM and BB conditions the differences in synchrony levels between real and pseudo dyads did not reach significance. These results suggest that even though participants are able to synchronize with each other in performing complex and unstructured movements (as evidenced by the high synchrony during their performance in the IS condition), they are not likely to synchronize behaviorally with each other spontaneously in this task. Thus, it is possible that when engaging in a motor action that requires cognitive effort, motor planning and complexity, synchronizing becomes more difficult, and therefore people are less likely to show noticeable spontaneous behavioral synchrony, in contrast to people's tendency to spontaneously synchronize during simple rhythmic and repetitive movements (Repp, 2005; Zivotofsky & Hausdorff, 2007).

Next, we examined changes in between and within‐brain coupling between participants while performing the task. As with the behavioral analysis mentioned above, we also compared the neural activation of real dyads, who performed the task together, with “pseudo dyads,” to rule out the possibility that the between‐brain synchrony reflected nonspecific brain activation related to the fact that all participants were performing a similar task, irrespective of interpersonal engagement. Results indicate greater between‐brain coupling in the L.IFG–L.IFG and dmPFC–dmPFC of “real dyads” compared with “pseudo dyads,” in both the FM and IS conditions, confirming that between‐brain coupling depends on real social engagement between participants. Between‐brain coupling in the baseline BB condition did not differ from the between‐brain coupling found in pseudo dyads, further confirming that the between‐brain coupling found in the IS and FM conditions relates to mutual attempts to coordinate behavior in these conditions. It seems that participants were attempting to synchronize spontaneously in the FM condition, as evidenced by a higher than baseline between‐brain coupling in that condition, but given the complexity of the 3D movements they did not achieve behavioral synchronization.

Moreover, we found greater between‐brain coupling in the R.IFG–R.IFG in the IS condition compared with the BB condition (in Real groups), indicating that between‐brain coupling in the IS condition is high and different from a condition with no face‐to‐face interaction. In line with this, greater activity in the right IFG was found during mutual execution of a motor act compared with performing the same motor act alone (Bhat et al., 2017), and greater coupling of the right IFG was found to be associated with joint attention (Saito et al., 2010) and non‐verbal coordination (Minagawa et al., 2018). Moreover, a previous fMRI study suggested a positive correlation between activity in the right IFG and the degree to which one's own actions depended on and needed to be temporally coordinated with the actions of another individual to successfully achieve a shared goal (Newman‐Norlund et al., 2008). The dmPFC is also strongly related to mentalizing processes, as part of the Theory of Mind network, which is reliably activated by diverse mentalizing paradigms (Lieberman, 2010). Likewise, the dmPFC was shown to be involved in monitoring others' actions and one's own social responses (Saxe, 2006). Specifically, previous studies emphasize the role of the dmPFC in error detection during interpersonal action coordination. It was shown that being “out of sync” during a dynamic joint action with a virtual partner is correlated with activation of dmPFC (Fairhurst et al., 2013) and activity in the dmPFC is associated with thinking about dissimilar others (Frith & Frith, 2006).

We also found greater between‐brain coupling between participants during the IS condition compared with the BB condition in the dmPFC–dmPFC ROI. Given the role of the dmPFC in monitoring and error detection, it is likely that the participants were coordinating their predictions of the other's movement. This suggests that intentional synchronization requires enhanced coordinated monitoring of the other's actions. It should be noted that the observed greater between‐brain coupling between participants in the “real dyads” may reflect the common cognitive processing, which participants share while engaging in an act of motor movement during the IS condition (Hamilton, 2020).

Increased between‐brain coupling emerging even in the absence of behavioral synchrony may indicate that between‐brain coupling is not an epiphenomena of doing the same thing at the same time and may represent preparation for synchronization. That is, between‐brain synchrony may support preparedness for behavioral synchrony.

However, since participants in the FM and IS conditions shared sensory input and motor output, but only during the IS condition were highly synchronized, it is more likely that the greater between‐brain synchrony in the IS condition represents joint activation related to the ongoing mutual adaptation within dyads, which is specific to synchrony. This assumption is further supported by the correlation between between‐brain coupling and task performance in this study. Interestingly, we also found increase in between‐brain coupling in one ROI (dmPFC–dmPFC) in the back‐to‐back condition in real versus pseudo‐pairs. Between‐brain coupling in the absence of real face‐to‐face interaction could be explained by the shared environment that includes odors and audio signals as well as preparation for synchronization. This suggests that between‐brain networks contribute to effective performance in the IS condition such that coordinated predictions regarding the other action is critical for synchronization.

Notably, analysis of within‐brain coupling indicated greater within‐brain coupling in both the FM and IS conditions, indicating that within‐brain coupling between the observation‐execution and monitoring system is increased during social interactions. Nonetheless, while we found that between‐brain coupling is critical for intentional synchronization, within‐brain coupling predicted synchronization during free movement. It appears that when participants were explicitly instructed to synchronize their behavior, they generally succeeded in doing so, and this synchrony is increased when between‐brain in general increases—that is, when gaps are detected and corrected in a coupled manner. However, during free movement when there is high self‐focus on one's own movement, large gaps may develop in behavior, leading to high within‐brain coupling. Intriguingly, within‐brain coupling negatively predicted intentional synchronization, suggesting that reliance on within‐brain communication between the error monitoring and observation–execution systems actually weakens behavioral synchronization. It is possible that during the initial phase of attempting to synchronize in the FM condition, when there is a large gap between the participants, coordination between these networks within one's brain is critical. However, when synchronization is high in the IS condition, it becomes more effective to rely on the others' predictions and movement direction as opposed to one's own representation of action and prediction. Notably, Koban et al. (2017) have recently argued that synchronized neural representations between interacting partners correspond with the brain's broad tendency to reduce prediction errors. The authors suggest that the error detection system (including the dmPFC) should increase its activity when there is a large gap between participants (low synchrony) and decrease its activity when there is no gap (high synchrony). Thus, the transition from reliance on within‐brain connections to between‐brain connections could point to a shift from a within‐brain feedback loop to a two‐brains feedback loop during interpersonal synchrony, whereby a dynamic mutual alignment is obtained by coordinating between the error monitoring system and observation–execution systems between interacting partners. As noted above, such balance between within‐brain and between‐brain networks is found in similar topological dynamics that change from segregation to integration between brain networks (Deco et al., 2015; Park & Friston, 2013; Tononi et al., 1994).

## 6 LIMITATIONS

Further investigation of the dynamics of behavioral synchrony, between‐brains and within brain coupling, is required. In particular, although the results of the current work showed that instructed synchrony is predicted by between‐brain coupling while within‐brain coupling negatively predicts behavior, these results cannot be interpreted as showing causal relationship between brain and behavior. The findings are suggestive and may pave the way for future examinations with larger samples, balanced samples of men and women, and additional fNIRS channels that will allow examination of larger scale network reconfiguration.

In conclusion, this study demonstrates that the brain has the ability to change its functional network organization of between‐ and within‐brain networks engaged by different task conditions.

The findings that between‐brain coupling positively predicts intentional synchrony while within‐brain coupling negatively predicts intentional synchrony and that within‐brain coupling predicts synchronization during free movement, may indicate that within‐brain coupling is important for the early phases of attempting to synchronize (free movement) but when synchronization is obtained there is a growing need for reliance on between‐brain networks. This emphasizes the utility of interpreting task related differences in brain organization in terms of within‐ and between‐brains organization. We hold that probing the balance between networks may elucidate the optimal network structures underlying various aspects of effective social interactions. One limitation of the current study is the absence of short separation channels (SSCs) in the systems we used. This caused the measured fNIRS signals representing cerebral blood flow to be intermixed with blood flow to the scalp and other intermittent tissue. However, we counteracted this to the best of our ability by removing this interference in signal post‐processing by the method suggested by Zhang et al., 2016. Future research investigating the mechanisms underlying various types of social interactions may gain insight from network neuroscience approaches (Bassett et al., 2018) to further examine how the relationship between within‐ and between‐brain reconfiguration underlies successful communication.

## Data Availability

The data that support the findings of this study are available from the corresponding author upon reasonable request.

## Footnotes

^1^
Video of the Razer Motion Task is available using the following URL: https://youtu.be/VEPWVY9dzRM

^2^

https://www.mathgoodies.com/calculators/random_no_custom
